# Sub-Inhibitory Concentrations of Oxacillin, but Not Clindamycin, Linezolid, or Tigecycline, Decrease Staphylococcal Phenol-Soluble Modulin Expression in Community-Acquired Methicillin-Resistant Staphylococcus aureus

**DOI:** 10.1128/spectrum.00808-21

**Published:** 2022-01-19

**Authors:** Elisabeth Hodille, Laetitia Beraud, Séverine Périan, Valentine Berti, Michèle Bes, Anne Tristan, Emilie Blond, Gérard Lina, Oana Dumitrescu

**Affiliations:** a Department of Bacteriology, Hospices Civils de Lyon, Hôpital de la Croix-Rousse, Centre de Biologie Nord, Lyon, France; b Centre International de Recherche en Infectiologie (CIRI), INSERM U1111, CNRS UMR5308, ENS Lyon, Université Lyon 1, Lyon, France; c Institut de Génomique Fonctionnelle de Lyon, Lyon, France; d Assistance Publique des Hôpitaux de Paris, Paris, France; e National Reference Center for Staphylococci, Hospices Civils de Lyon, Hôpital de la Croix-Rousse, Centre de Biologie Nord, Lyon, France; f Department of Biochemistry and of Molecular Biology, Hospices Civils de Lyon, Centre Hospitalier Lyon Sud, Centre de Biologie Sud, Lyon, France; Riverside University Health System, Medical Center -University of California

**Keywords:** *Staphylococcus aureus*, community-acquired methicillin-resistant *Staphylococcus aureus*, ST8 USA300 clone, ST80 European clone, virulence factors, phenol-soluble modulins alpha, delta-hemolysin, antibiotics, subinhibitory concentration, modulation expression

## Abstract

Staphylococcus aureus (SA) is a major human pathogen producing virulence factors, such as Panton-Valentine-leucocidin (PVL), alpha-hemolysin (Hla), and phenol-soluble-modulins alpha (PSMα), including delta-hemolysin (Hld). Unlike oxacillin, clindamycin and linezolid subinhibitory concentrations (sub-MIC) display an anti-toxin effect on PVL and Hla expression. Few studies have investigated PSMα and Hld expression modulation by antibiotics. Herein, we assessed the effect of antibiotic sub-MIC on PSMα1 and Hld expression for 4 community-acquired methicillin-resistant SA (CA-MRSA), 2 strains belonging to USASA300 and 2 strains belonging to ST80 European clone. SA were grown under oxacillin, clindamycin, linezolid, or tigecycline. After incubation, culture pellets were used for the determination of *psmα1*, *pmtB*, *pmtR* mRNA, and *RNAIII* levels by relative quantitative RT-PCR. PSMα1 and Hld expressions were measured in supernatant using high-performance-liquid-chromatography coupled to mass-spectrometry (HPLC-MS). Oxacillin sub-MIC reduced PSMα1 and Hld production, partially related to mRNA variations. For other antibiotics, effects on toxin expression were strain or clone dependent. Antibiotic effect on mRNA did not always reflect protein expression modulation. Variations of *pmtB*, *pmtR* mRNA, and RNAIII levels were insufficient to explain toxin expression modulation. Altogether, these data indicate that PSMα and Hld expressions are modulated by antibiotics (potential anti-toxin effect of oxacillin) differently compared to PVL and Hla.

**IMPORTANCE** Staphylococcal toxins play an important role in the physiopathology of staphylococcal infections. Subinhibitory concentrations (sub-MIC) of antibiotics modulate *in vitro* toxins expression in S. aureus: clindamycin (CLI) and linezolid (LIN) display an anti-toxin effect on Panton-Valentine leucocidin and alpha-hemolysin production, while oxacillin (OXA) has an inducing effect. Few studies have focused on the modulation of phenol-soluble modulins alpha (PSMα) including delta-hemolysin expression by sub-MIC antibiotics. The aim of the present study was to investigate the effects of sub-MIC antibiotics on the expression of PSMα toxins for 4 community-acquired methicillin-resistant *S. aureus* (CA-MRSA) clinical isolates. The data presented herein confirm that OXA sub-MICs constantly inhibit PSMα production for CA-MRSA. Certain strains of S. aureus are highly sensitive to sub-MICs of protein synthesis inhibitory agents, resulting in an important increase of mRNA levels to overcome the intrinsic ribosome blockage ability of these antibiotics, eventually translating in increased expression of toxins.

## INTRODUCTION

Staphylococcus aureus is a pathogen responsible for diverse infections, such as severe skin and soft tissue infection (SSTI), life-threatening necrotizing pneumonia, and bacteremia (1). Staphylococcal toxins such as Panton-Valentine leucocidin (PVL), alpha-hemolysin (Hla), and phenol-soluble modulins alpha (PSMα), including delta-hemolysin (Hld), play an important role in the physiopathology of these infections (2–6). It is well known that subinhibitory concentrations (sub-MIC) of antibiotics modulate *in vitro* toxin expression in S. aureus: clindamycin (CLI) and linezolid (LIN) display an anti-toxin effect on PVL (7–9) and Hla (7, 9–13) production, while oxacillin (OXA) has an inducing effect (7, 14–20). Moreover, experiments on animal models support the use of protein-synthesis inhibitor antibiotics such as CLI or LIN, rather than vancomycin, for a better outcome in methicillin-resistant S. aureus (MRSA) necrotizing pneumonia (21, 22). Thus, some guidelines empirically recommend using anti-toxin drugs (CLI or LIN) for the management of toxin-mediated staphylococcal infections (23). The modulation of virulence factor expression by sub-MIC of antibiotics involves several pathways depending on the molecule considered. For example, LIN and CLI act mainly by blocking ribosomal function and inhibiting the protein synthesis of virulence factors or of their regulatory system (24). Alternatively, OXA induces toxin gene transcription by triggering the SOS response in bacteria or by interfering with regulatory networks (notably with the overall staphylococcal regulators SarA, MgrA, and ArlR) that govern virulence expression (24). Furthermore, few studies have focused on the modulation of PSMα expression by sub-MIC antibiotics, and their results are inconsistent. An overall anti-toxin effect has been observed for OXA (20, 25) but the impact was variable for CLI and LIN (25–27). These authors have suggested that the modulatory effects may involve the Agr system, the main overall regulator of toxin production of S. aureus (28), since PSM expression depends on the activation of the transcriptional factor AgrA while Hld is encoded by RNAIII. Nevertheless, alterations in the Agr system did not recapitulate all the expected phenotypes, indicating that the underlying mechanisms may be more complex (24). PSMα extracellular export requires a special transporter, Pmt, composed of 4 subunit (ABCD) (29), whose expression is regulated by the repressor pmtR (30). PSM produced during bacteria growth bind to repressor pmtR, allowing *pmtA*, *pmtB*, *pmtC*, *pmtD*, and *pmtR* transcription (30).

The aim of the present study was to investigate the effects of sub-MIC of 4 anti-staphylococcal antibiotics (CLI, LIN, OXA, and tigecycline [TIG]) on PSMα1 and Hld expression. To do so, we used 4 community-acquired MRSA (CA-MRSA) clinical isolates belonging to 2 worldwide spreading lineages (ST8 USA300 and ST80 European). Moreover, we assessed the impact of antibiotics on the expression of the essential transport system via *pmtB* and *pmtR* mRNA levels.

## RESULTS

### Minimum inhibitory-concentrations (MICs) determination.

The MICs of CLI, LIN, OXA, and TIG were determined by microdilution method using Brain-Heart infusion broth (Table 1).

**TABLE 1:** MICs of antibiotics and toxins production level for the S. aureus isolates^*a*^

Strains	Clones	MIC (mg/liter) in BHI broth				Toxin production (μg/mL) without antibiotics at 24-h incubation (mean ± SD)	
		CLI	LIN	OXA	TIG	Hld	PSMα1
HT20060752	USA300	0.125	2	32	2	137.0 **±** 4.7	49.9 **±** 4.6
SF8300	USA300	0.25	2	32	4	135.1 **±** 9.8	40.6 **±** 2.4
LUG1799T	ST80 European	0.125	0.5	16	2	120.6 **±** 2.6	16.2 **±** 0.03
ST20121288	ST80 European	0.125	1	16	1	108.9 **±** 0.4	13.8 **±** 1.2

a CLI: clindamycin, LIN: linezolid, OXA: oxacillin, TIG: tigecycline, BHI: brain heart infusion, Hld: delta-hemolysin, PSMα1: phenol-soluble modulins alpha 1, SD: standard deviation.

### Effect of antibiotics on Hld production.

**FIG 1:**
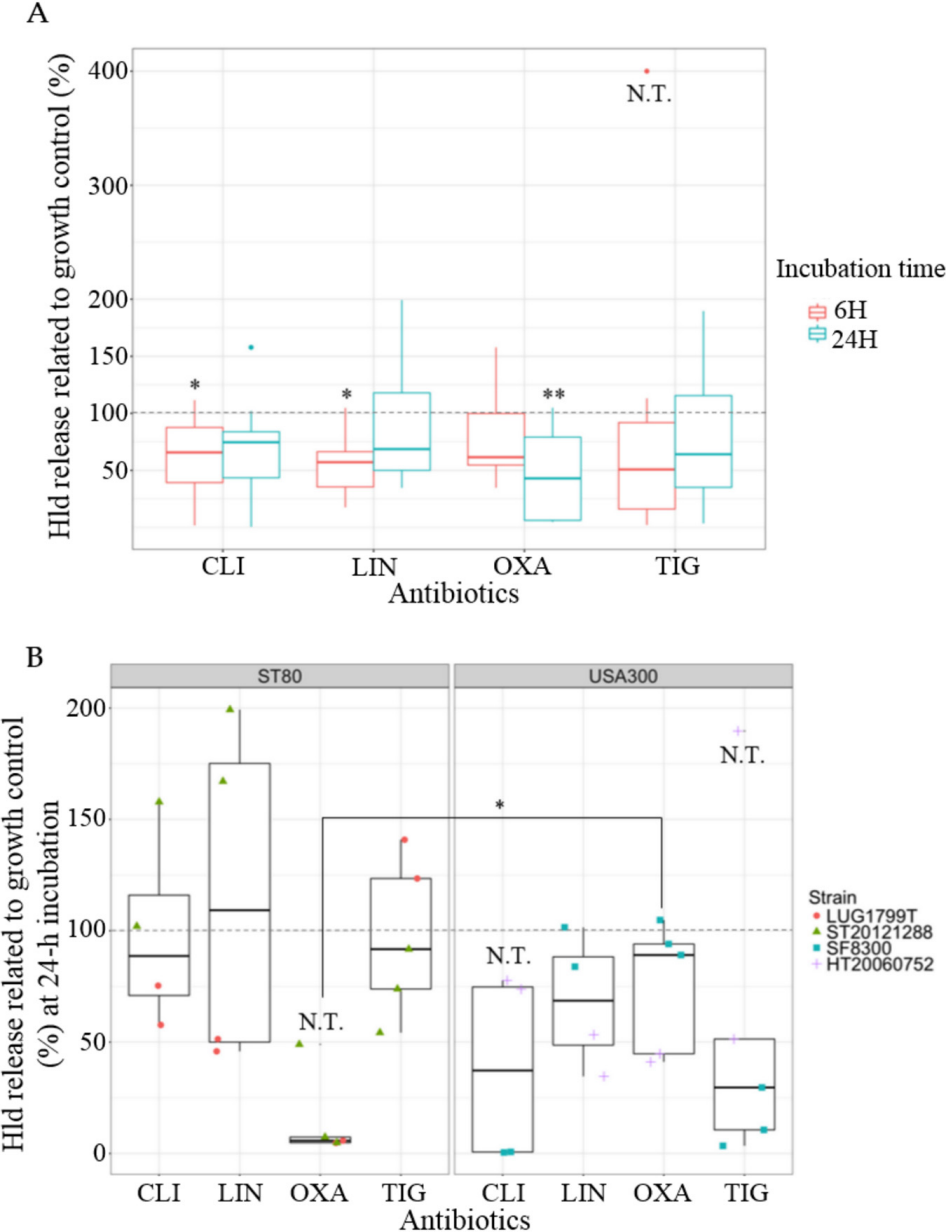
Effects of antibiotic sub-MIC on Hld release. (A) Hld release at 6-h or 24-h incubation, pooling the 4 community-acquired methicillin-resistant S. aureus. (B) Hld release at 24-h incubation according to the clones (ST80 European clone on the left, ST8 USA300 on the right), pooling the different sub-MICs for one antibiotic. The results are expressed in percentage related to growth control without antibiotic (= 100%) represented by the dotted gray line. When possible, conformity t tests were performed to compare the expression with and without antibiotic. If not, N.T. is indicated. Comparison of Hld release between both clones for OXA condition was performed using the nonparametric Wilcoxon test. (A) CLI: clindamycin (*n* = 8), LIN: linezolid (*n* = 8), OXA: oxacillin (*n* = 10), TIG: tigecycline (*n* = 10). (B) 1 point corresponds to 1 value (orange circle for LUG1799T; green triangle for ST20121288; blue square for SF8300; purple cross for HT20060752). * *P* < 0.05, ** *P* < 0.01, N.T.: not testable.

Considering all 4 strains together, CLI and LIN sub-MIC resulted in a significant decrease of Hld production compared to the growth control at 6-h incubation, not observed at 24-h incubation (Fig. 1A). On the contrary, OXA sub-MIC induced a time-dependent decrease of Hld release (Fig. 1A). The decrease was nonsignificant at 6-h, but became significant at 24-h incubation reaching 44.55% (95%CI, confidence interval [16.18; 72.92]) of Hld released compared to growth control.

When analyzing the result strain by strain, the effects of CLI and LIN sub-MIC on Hld production were strain specific. With CLI sub-MIC, a dramatic Hld decrease was observed only for SF8300 at 6-h and 24-h incubation (Fig. S1A, Fig. 1B), while a slight reduction (LUG1799T, HT20060752) or induction (ST20121288) of Hld expression were observed for the other CA-MRSA at 24-h incubation (Fig. 1B). LIN sub-MIC resulted in an increase of Hld release for ST20121288 at 24-h incubation, while no effect (SF8300) or a slight inhibitor effect (LUG1799T, HT20060752) was observed for the other CA-MRSA (Fig. 1B).

The effect of OXA and TIG were rather clone dependent. OXA inhibitory effect on Hld release was significantly stronger for ST80 European clone compared to ST8 USA300 at 6-h and 24-h incubation (Fig. S1A and Fig. 1B): 49.15% (95%CI [32.39; 65.91]) at 6-h and 14.33% (95%CI [9.76; 38.42]) at 24-h of Hld release compared to growth control for ST80 European, while no significant difference of Hld release was observed for ST8 USA300 clone compared to the growth control for both time points. With TIG sub-MIC, a nonsignificant trend toward inhibition of Hld release (mean at 56.95% compared to growth control) was observed for ST8 USA300 clone, while Hld release was unmodified for ST80 European clone (mean at 98.85%, Fig. 1B). The nonsignificance could be due to the presence of an outlier for HT20060752 belonging to the ST8 USA300 clone.

### Effect of antibiotics on PSMα1 production.

**FIG 2:**
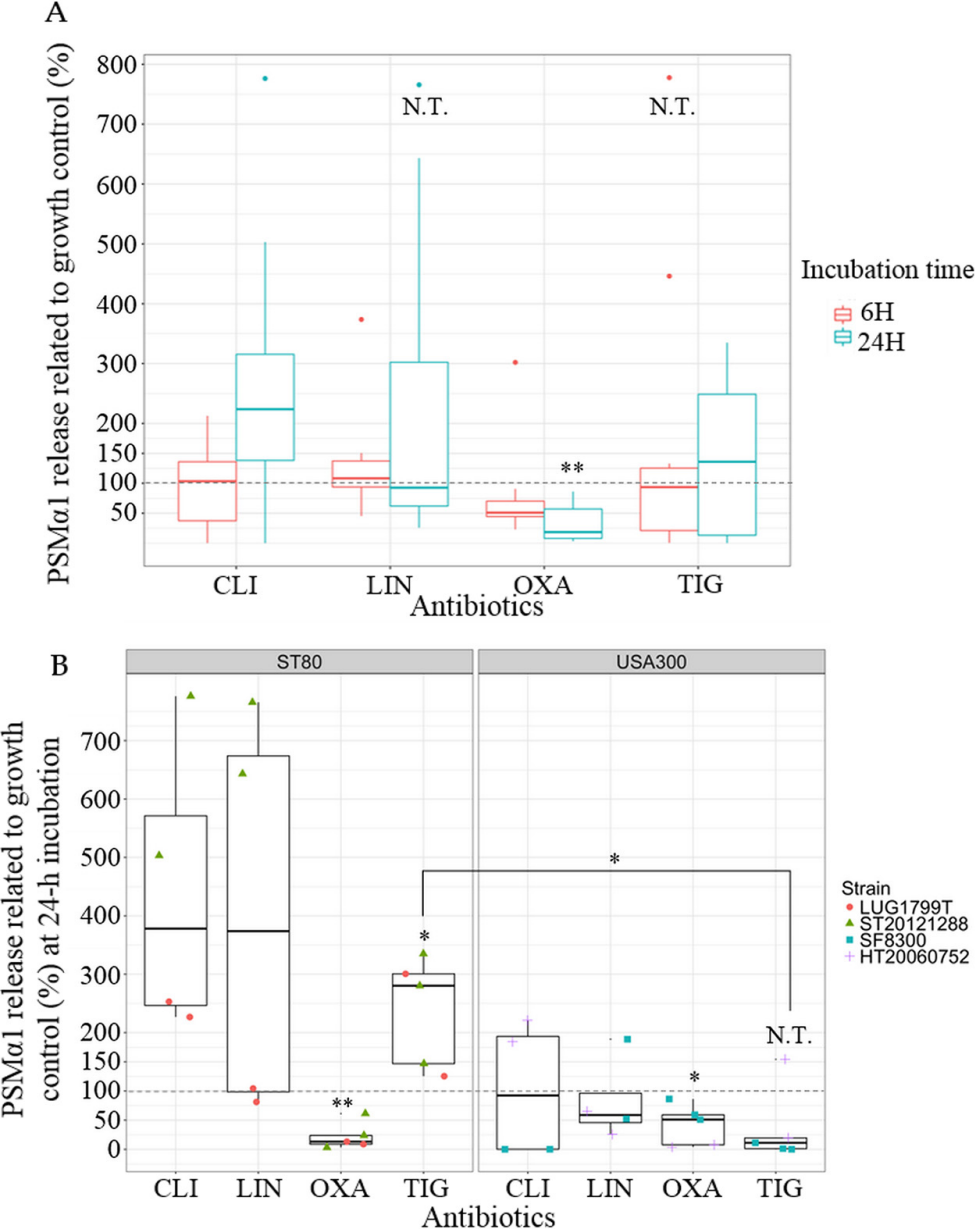
Effects of antibiotic sub-MIC on PSMα1 release. (A) PSMα1 release at 6-h or 24-h incubation, pooling the 4 community-acquired methicillin-resistant S. aureus. (B) PSMα1 release at 24-h incubation according to the clones (ST80 European clone on the left, ST8 USA300 on the right), pooling the different sub-MICs for one antibiotic. The results are expressed in percentage related to growth control without antibiotic (= 100%) represented by the dotted gray line. When possible, conformity t tests were performed to compare the expression with and without antibiotic. If not, N.T. is indicated. Comparison of PSMα1 release between both clones for TIG condition was performed using the nonparametric Wilcoxon test. (A) CLI: clindamycin (*n* = 8), LIN: linezolid (*n* = 8), OXA: oxacillin (*n* = 10), TIG: tigecycline (*n* = 10). (B) 1 point corresponds to 1 value (orange circle for LUG1799T; green triangle for ST20121288; blue square for SF8300; purple cross for HT20060752). * *P* < 0.05, ** *P* < 0.01, N.T.: not testable.

Considering all 4 strains together, only OXA sub-MIC resulted in a significant overall effect: a time-dependent decrease of PSMα1 release, nonsignificant at 6-h, but significant at 24-h incubation reaching 31.83% (95%CI [10.40; 53.26]) of PSMα1 release compared to growth control (Fig. 2A). Contrary on Hld release, PSMα1 release was significantly decreased for both clones at 24-h incubation (Fig. 2B).

When analyzing the result strain by strain, CLI and LIN sub-MIC resulted in a strain-specific effect. With CLI treatment, a dramatic PSMα1 decrease was observed for SF8300 at 6-h and 24-h incubation (Fig. S1B, Fig. 2B), while there was a trend toward increased PSMα1 expression (Fig. 2B) for the other CA-MRSA at 24-h incubation. With LIN treatment, an important increase of PSMα1 release at 24-h incubation was observed for ST20121288 only, while no effect (LUG1799T), slight inhibitor effect (HT20060752), or discrepant effect (SF8300) was observed for the other CA-MRSA (Fig. 2B).

TIG effects on PSMα1 release appeared to be clone-dependent (Fig. 2B), despite the presence of an outlier for HT20060752. At 24-h TIG incubation, PSMα1 release was significantly higher for ST80 European clone (237.57%; 95%CI [119.54; 355.61]) compared to ST8 USA300 clone (37.29%; 95%CI [-44.50; 119.1]).

In order to investigate the hypothesis of a transcriptional origin for the observed modulation in toxin levels, toxin mRNA (RNAIII for *hld* and *psmα1* mRNA) levels were quantified by quantitative RT-PCR.

### Effect of antibiotics on RNAIII.

**FIG 3:**
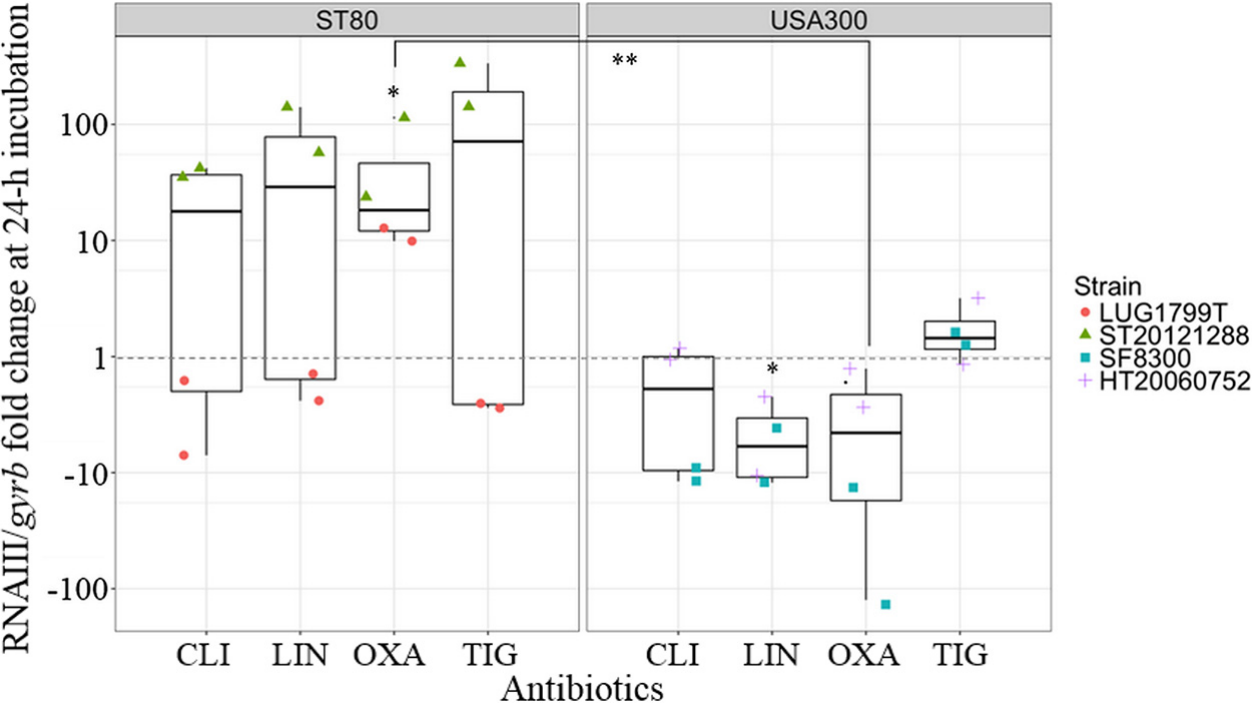
Effects of antibiotic sub-MIC on RNAIII expression at 24-h incubation according to the clones. The results for each antibiotic corresponding to different sub-MICs were pooled for the 2 strains belonging to the same genetic background, ST80 European clone on the left and USA300 clone on the right. The results are expressed as n-fold differences in the RNAIII/*gyrb* ratio in the presence of antibiotics relative to the control growth condition (without antibiotics, = 1) represented by the dotted gray line. Conformity t tests were performed to compare the expression with and without antibiotic. Comparison of RNAIII/*gyrb* fold change between both clones for OXA condition was performed using the Welch test. 1 point corresponds to 1 value (orange circle for LUG1799T; green triangle for ST20121288; blue square for SF8300; purple cross for HT20060752). CLI: clindamycin (*n* = 4), LIN: linezolid (*n* = 4), OXA: oxacillin (*n* = 4), TIG: tigecycline (*n* = 4). * *P* < 0.05, ** *P* < 0.01.

CLI, LIN, and TIG differently affected RNAIII according to the strain, resulting in a great increase only for ST20121288 belonging to the ST80 European clone. For the other strain of this clone, LUG1799T, RNAIII level was unmodified or slightly decreased. For ST8 USA300 clone, LIN treatment resulted in a significant decrease of RNAIII, CLI resulted in a RNAIII decrease only for SF8300, while TIG had not effect. With OXA treatment, we observed a clone-dependent behavior at 24-h incubation with a significant increase of RNAIII levels for ST80 European isolates and a trend toward a decrease (not significant) for ST8 USA300 isolates, but with a significant difference between both clones (Fig. 3).

### Effect of antibiotics on *psmα1* mRNA level.

**FIG 4:**
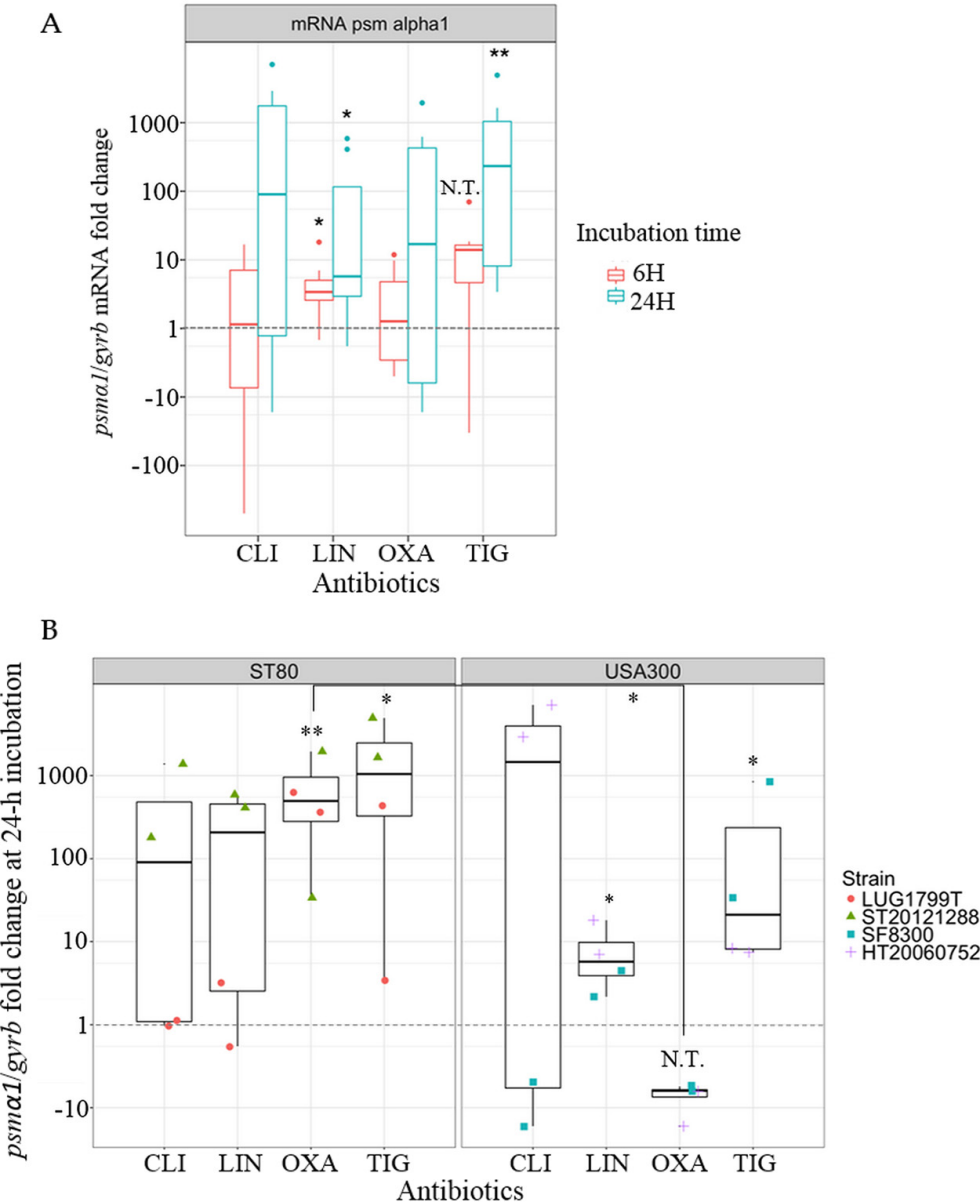
Effects of antibiotic sub-MIC on psmα1 mRNA levels. (A) psmα1 mRNA pooling the 4 community-acquired methicillin-resistant S. aureus at 6-h and 24-h incubation. (B) psmα1 mRNA at 24-h incubation according to the clones (ST80 European clone on the left, ST8 USA300 on the right), pooling the different sub-MICs for one antibiotic. The results are expressed as n-fold differences in the *psmα1*/*gyrb* ratio in the presence of antibiotics relative to the control growth condition (i.e., without antibiotics, = 1) represented by the dotted gray line. When possible, conformity t tests were performed to compare the expression with and without antibiotic. If not, N.T. is indicated. Comparison of *psmα1*/*gyrb* fold change between both clones for OXA condition was performed using the nonparametric Wilcoxon test. (A) CLI: clindamycin (*n* = 8), LIN: linezolid (*n* = 8), OXA: oxacillin (*n* = 8), TIG: tigecycline (*n* = 8). (B) 1 point corresponds to 1 value (orange circle for LUG1799T; green triangle for ST20121288; blue square for SF8300; purple cross for HT20060752). * *P* < 0.05, ** *P* < 0.01, N.T.: not testable.

Considering all 4 strains together, only LIN and TIG resulted in an overall significant increase of *psmα1* mRNA level, at 6-h and 24-h incubation for LIN and at 24-h incubation for TIG (Fig. 4A). In details, at 24-h incubation, LIN- and TIG-induced increase was significant for the ST8 USA300 clone. For the ST80 European clone, the increase was significant after TIG treatment, and especially marked after LIN treatment for ST20121288 (Fig. 4B).

With CLI, we noted a strain-dependent effect: a great decrease of *psmα1* mRNA level at 6-h (Fig. S2) and 24-h incubation (Fig. 4B) was observed for SF8300, while *psmα1* mRNA level was unmodified for LUG1799T, and strongly increased for HT20060752 and ST20121288, notably at 24-h incubation (Fig. 4B). With OXA, we observed a clone-dependent behavior at 24-h incubation with a significant increase of *psmα1* mRNA levels for ST80 European isolates and a trend toward a decrease (not testable) for ST8 USA300 isolates (Fig. 4B), thus differentiating significantly both clones.

**FIG 5:**
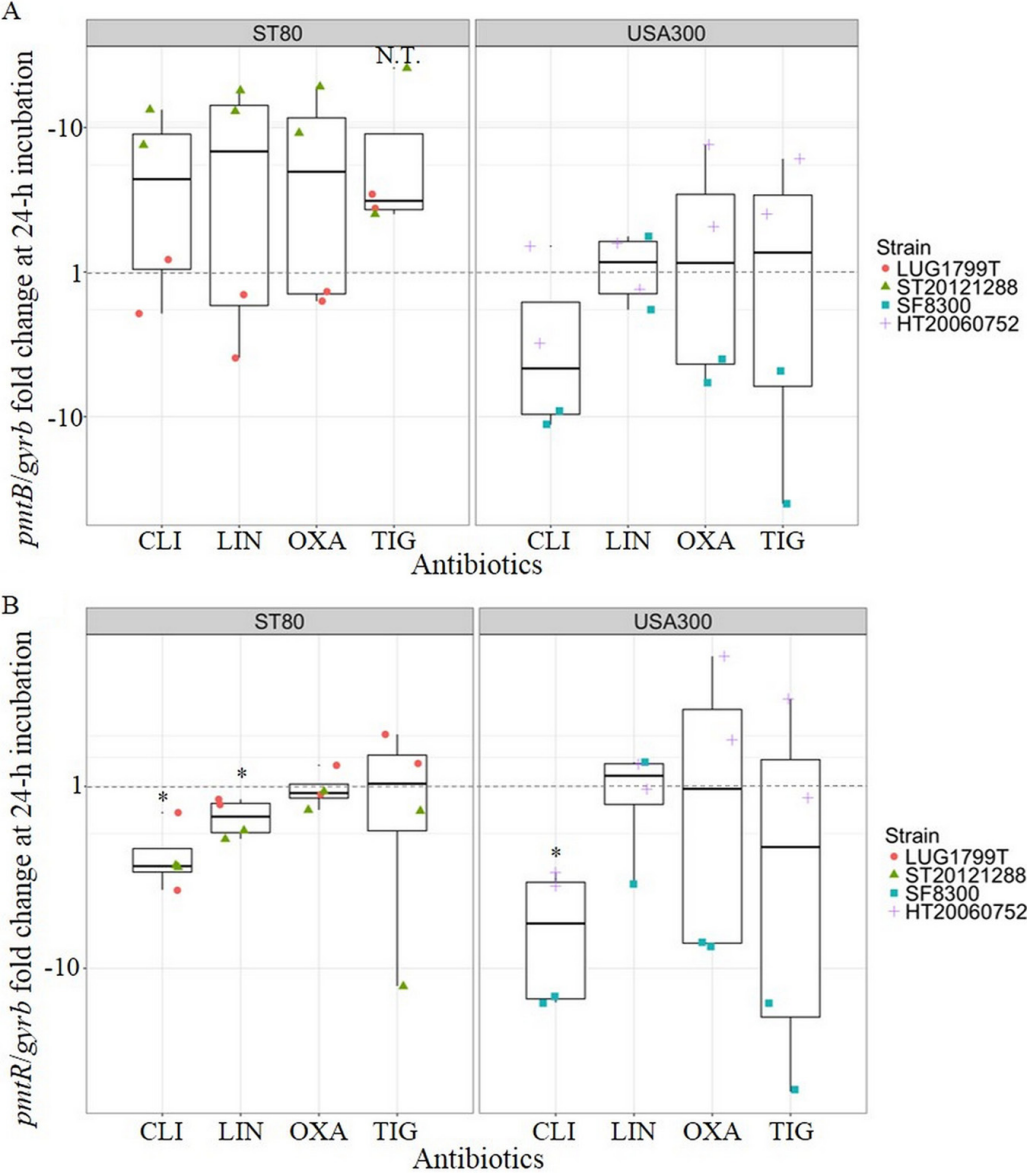
Effects of antibiotic sub-MIC on *pmtB* mRNA (A) and *pmtR* mRNA (B) expression at 24-h incubation according to the clones. The results for each antibiotic corresponding to different sub-MICs were pooled for the 2 strains belonging to the same genetic background, ST80 European clone on the left and USA300 clone on the right. The results are expressed as n-fold differences in the *pmtB/gyrb* or *pmtR/gyrb* ratio in the presence of antibiotics relative to the growth control condition (without antibiotics, = 1) represented by the dotted gray line. When possible, conformity t tests were performed to compare the expression with and without antibiotic. If not, N.T. is indicated. 1 point corresponds to 1 value (orange circle for LUG1799T; green triangle for ST20121288; blue square for SF8300; purple cross for HT20060752). CLI: clindamycin (*n* = 4), LIN: linezolid (*n* = 4), OXA: oxacillin (*n* = 4), TIG: tigecycline (*n* = 4). * *P* < 0.05, N.T.: not testable.

In order to investigate the mechanism underlying PSMα and Hld variations resulting from antibiotic sub-MIC treatment, we assessed the modulation by antibiotics of the PSM transporter expression, Pmt, through *pmtB* mRNA level (Fig. 5A), and of its natural repressor, *pmtR* (Fig. 5B).

### Effect of antibiotics on *pmtB*, *pmtR* mRNA.

Regarding the ST80 European clone, we observed a significant decrease of *pmtR* mRNA levels (Fig. 5B) for CLI and LIN sub-MIC treatment, while *pmtB* mRNA levels (Fig. 5A) were slightly modified for LUG1799T and significantly increased for ST20121288. *PmtB* mRNA levels (Fig. 5A) were clearly increased by OXA treatment for ST20121288 (around 10-fold compared to growth control) but unmodified for LUG1799T, while *pmtR* mRNA level (Fig. 5B) was unmodified for both strains. *PmtB* and *pmt*R mRNA levels (Fig. 5) were not significantly modified after TIG treatment.

For the USA300 clone, CLI treatment significantly decreased *pmtR* mRNA level (Fig. 5B). For *pmtB* mRNA (Fig. 5A), the decrease was observed only for the SF8300 strain. With LIN, *pmtB* and *pmtR* mRNA levels were unmodified. After OXA treatment, *pmtB* and *pmtR* mRNA mean levels appeared not significantly modified. However, these were rather decreased for SF8300 and increased for HT20060752. TIG treatment strongly decreased *pmtB* and *pmtR* mRNA levels for the SF8300 strain (inferior to 10-fold change), while these were unmodified or slightly increased for HT20060752.

## DISCUSSION

Previous studies have reported an inhibitory effect of OXA sub-MIC on PSMα and Hld production (20, 25), which is consistent with the findings reported herein. Although the tested conditions were different in the study of Joo et al. (25) (LAC strain, different from those tested here but also belonging to the USA300 lineage; antibiotic used at 1/50 MIC), these results were concordant with our observations, since PSMα1 expression was decreased by OXA treatment at 24-h incubation. These observations were however different from other reports of other staphylococcal virulence determinants modulated by OXA, such as the increase of PVL and Hla expression (7, 14–19). It has been shown that the mechanism underlying the increased expression of PVL upon OXA treatment involved an activation of *pvl* transcription, consecutive to the activation of Staphylococcal overall regulators (*SarA*, *Mgr*, *Arl*) (24). Herein, we observed that for the ST80 European isolates, *psmα1* mRNA expressions were largely and significantly upregulated at 24-h incubation with OXA sub-MIC. This increase resulted probably from an activation of the overall regulator Agr, since RNAIII was also significantly upregulated. Nevertheless, the ultimate decrease of PSMα1 and Hld expression may indicate a blockage of either the toxin production occurring at the translational level, or a blockage of the toxin secretion, through a possible downregulation of Pmt transporter (29). *pmtB* mRNA level was unmodified or even increased for the ST80 European strains, suggesting that the PSM secretion system was not repressed by OXA treatment and that would unlikely be the cause of PSM decreased expression. Therefore, this possibly points toward the blockage of *psmα1* mRNA and RNAIII translation by OXA, according to an unknown mechanism. Further studies will be necessary to investigate this point.

For the USA300 isolates, the inhibition of Hld expression induced by OXA was lower than for the ST80 European isolates, while the RNAIII expression tended to decrease for the USA300 isolates. This could indicate that toxin decrease by OXA relies on different mechanisms depending on the clones. For the USA300 clone, we can hypothesize that the inhibition of PSM and Hld expression relies on a decreased transcription consecutive to Agr repression. Though decreased *psmα1* mRNA after OXA treatment was not described by Joo et al. for the LAC strain (25), this could result from different experimental settings. Indeed, in the study of Joo et al., S. aureus LAC was incubated with OXA for 8 h while in our study, decreased *psmα* mRNA level upon OXA treatment was observed at 24-h incubation but not at 6-h incubation.

Recently, Bojer et al. have showed that PSM expression limited persister cell populations in S. aureus (31). The authors determined the frequency of persisters after incubation with 20× MIC of ciprofloxacin, gentamicin, or OXA for S. aureus Newman wild-type strain (WT) or its isogenic PSM mutant (ΔPSMαβhld); this frequency was significantly lower for WT strain than for ΔPSMαβhld strain under ciprofloxacin and gentamicin treatment, indicating that the lack of PSM expression was associated with an increase of persister frequency. Interestingly, upon OXA treatment, the frequencies of persisters between the WT strain and ΔPSMαβhld strain was not significantly different, as the WT strain already displayed a very high frequency of persisters. The authors hypothesized that the tolerance of the WT strain to OXA treatment could be unrelated to PSM expression. Nevertheless, even if Bojer et al. have tested concentrations of antibiotics superior to MIC, our results could provide additional explanation: upon OXA treatment, the inhibition of PSM expression may be equivalent to the lack of PSM expression of the ΔPSMαβhld strain, both resulting in similar tolerant phenotypes.

For protein synthesis inhibitory agents, results were variable according to the clone or the strain. Interestingly, results of PSMα and Hld expression upon antibiotic exposure were discordant with previously published data supporting these agents as anti-toxin molecules, notably CLI and LIN as PVL (7–9) and Hla suppressive agents (7, 9–13). CLI treatment decreased PSMα1 and Hld production only for the SF8300 strain. For this strain, CLI led to a strong decrease of *psmα1* mRNA expression, and PSMα1 and Hld production. These results indicated a potential transcriptional underlying mechanism, since RNAIII level was also decreased, suggesting an overall repression of the Agr system. In addition, *pmtB* mRNA decreased level indicated a potential downregulation of transport of PSM. For other strains (the ST80 European isolates and the other USA300 strain [HT20060752]), CLI increased *psmα1* mRNA level and PSMα1 expression, in a variable manner according to strains (strong increase for ST20121288 related to great activation of Agr system, moderate increase for LUG1799T and HT20060752). Previous studies investigating CLI effects on PSM production were contradictory. While Joo et al. have showed an increase of PSMα1 production and *psmα* mRNA expression for LAC (25), concordant with the observed effects herein on the majority of tested strains, Yamaki et al. have concluded first about an inhibitory effect of CLI and TR-700 (tedizolide) on PSM production (26) and second about an inducer effect in another study (27). In conclusion, the effect of CLI on PSMα and Hld expression features strain-dependent profiles, relying on potentially multiple still unknown mechanisms, but display a predominant inducer activity.

Upon LIN exposure, a consistent effect of *psmα1* mRNA increased expression was observed for all CA-MRSA tested strains. We concomitantly observed a weak but significant decrease of RNAIII level for the USA300 clone, while a great increase of RNAIII level was shown for the ST20121288 strain, arguing for an important activation of the regulator Agr. Accumulation of toxin mRNA following LIN treatment was yet observed for *hla* mRNA in the study by Otto et al. (9). Indeed, LIN is a 50S ribosome-inhibitory agent blocking several steps of the translational process resulting in the accumulation of mRNA and other intermediate products of the translational complex (32). This could explain the increase of toxin mRNA amount observed in the USA300 clone despite Agr being repressed. Nevertheless, the effect thereafter on the production of toxins was strain dependent. Thus, LIN blocked effectively translation for all tested CA-MRSA strains, except for ST20121288 strain. For the latter strain, we could hypothesize that the accumulation of mRNA added to Agr regulator activation may foster mRNA translation overcoming the LIN ribosome-blockage capacity.

In our study, TIG treatment strongly induced *psmα1* mRNA expression for all tested CA-MRSA strains at 24-h incubation. Consistent Agr activation supported by increased RNAIII level upon TIG treatment was observed for one strain (ST20121288, belonging to the ST80 European clone). In contrast, TIG sub-MIC effect on PSMα1 and Hld expression was clone dependent. Indeed, we observed a significant difference between the USA300 clone and the ST80 European clone with an inhibitory effect on Hld production more pronounced for the USA300 clone than for the ST80 European clone. Moreover, TIG exposure displayed an inductor effect on PSMα1 expression for the ST80 European clone, and conversely an inhibitor effect for the USA300 clone. These observations were partly discordant with those of Yamaki et al., who have reported a constant inductor effect of TIG sub-MIC on PSM expression (27). Variation of *pmtB* expression induced by TIG may not completely explain the different strain-specific features regarding PSMα1 and Hld production. Although the pattern of *pmtB* expression, increased for the ST80 European clone and decreased for the SF8300 strain, was consistent with either a boosted transport or a blockage of PSM secretion, its expression was slightly increased for HT20060752 and inconsistent with the inhibitory effect observed with TIG for this last strain. Nevertheless, the ribosomal blocking function underlying the protein synthesis inhibitory ability of TIG (33) may prevail under certain circumstances, thus resulting in an overall inhibitory effect.

## CONCLUSIONS

In conclusion, our results confirm that OXA sub-MICs constantly inhibit PSMα production for CA-MRSA, despite different underlying mechanisms and genetic backgrounds. Hld is variably modulated by OXA according to the CA-MRSA clone. Certain strains of S. aureus (herein ST20121288) are highly sensitive to sub-MICs of protein synthesis inhibitory agents, resulting in an important increase of mRNA levels to overcome the intrinsic ribosome blockage ability of these antibiotics, eventually translating in increased expression of toxins. The modulation of PSMα and Hld production by antibiotic sub-MIC involve complex mechanisms, including the modulation of Agr system, *pmtB* expression, and undoubtingly other unknown mechanisms. The variations of toxin mRNA levels are not always consistent with protein levels, indicating that proteomic analyses are essential to conclude on the effective toxin modulation by sub-MICs of antibiotics.

Altogether, these data indicate that PSMα and Hld are modulated by antibiotics (potential anti-toxin effect of OXA) differently than other previously studied staphylococcal toxins (PVL and Hla). This could reveal benefic for the OXA-based management of acute staphylococcal infection, as PSM and Hld play an important role in pathogenesis.

## MATERIALS AND METHODS

### Bacterial strains.

Four S. aureus clinical isolates were selected for this study, representing major CA-MRSA clinical isolates spreading in community: 2 strains belonging to the CA-MRSA PVL+ USA300 clone and 2 strains to the CA-MRSA PVL+ European clone ST80 (Table 1).

### Antibiotics and MIC determination.

The antibiotics used in this study were CLI, LIN, OXA, and TIG; OXA was purchased from Sigma-Aldrich (L’Isle d’Abeau, France), other antibiotics were provided by Pfizer (Ambroise, France). MICs were determined by microdilution method using BHI broth (bioMérieux, Marcy l’Etoile, France).

### Bacterial culture.

Strains were cultured on Trypticase blood agar plate, which were incubated overnight at 37°C. Isolated colonies were resuspended in BHI broth and the suspension was adjusted to a turbidity equivalent to that of a 0.5 McFarland standard. Cultures were grown at 37°C under gyratory shaking (180 rpm). When the optical density (OD) reached a turbidity equivalent to that of a 1 McFarland standard, sub-MIC of antibiotics were added to the glass culture tubes. For each antibiotic and each strain, the sub-MIC was chosen as the one not inhibiting significantly bacterial growth (Fig. S3): LUG1799T was tested with 1/4 and 1/8 of CLI, LIN and OXA, and 1/8 and 1/16 of TIG; ST20121288 was tested with 1/4 and 1/8 of CLI, LIN and TIG, and 1/8 and 1/16 of OXA; SF8300 was tested with 1/4 and 1/8 of CLI and LIN, and 1/4, 1/8 and 1/16 of OXA and TIG; HT20060752was tested with 1/4 and 1/8 of CLI and LIN, 1/8 and 1/16 of OXA, and 1/16 and 1/32 of TIG. Cultures with or without antibiotics (growth control) were re-incubated at 37°C with shaking during 6-h and 24-h. Aliquots were sampled 6 and 24-h later.

### PSMα1 and Hld quantification.

After centrifugation of 24h-incubated aliquots at 4500 g for 10 min, bacterial supernatants were diluted 1/5 with methanol and incubated at 4°C for 15 min. Diluted supernatants were then centrifuged at 10000 g for 5 min and supernatants were recovered for PSMα1 and Hld quantification by high-performance liquid chromatography mass spectrometry (HPLC-MS) with an Agilent system using the method described elsewhere (34). PSMα1 and Hld quantifications for each condition were normalized related to AUC of growth curve (Fig. S3) to prevent any bias related to growth slowdown. The percentages of toxin release for each condition were calculated related to growth control without antibiotic according to formula: $\%=\frac{\left(\text{test with antibiotic})/(\text{AUC test}\right)}{(\text{growth control without antibiotic})/(\text{AUC control})\;}\;$ For information, PSMα1 and Hld release expressed as μg/ml for each strain under control condition (without antibiotics) at 24-h incubation were available in Table 1.

### Relative quantitative RT-PCR.

Aliquots of 2 ml of each culture were centrifuged at 4500 g for 10 min. The pellets were washed with 0.5 ml of Tris buffer (10 mM) and centrifuged at 4500 g for 10 min. The turbidity was then adjusted to a turbidity equivalent to that of a 3 McFarland standard. A 1.5 ml aliquot of turbidity-adjusted and washed bacterial suspension was centrifuged at 4500 g for 10 min, and the pellets were treated with lysostaphin (Sigma-Aldrich) at a final concentration of 200 μg/ml. The total RNA contained in the pellets was then extracted and purified using the Qiagen RNeasy Plus minikit (Qiagen, Hilden, Deutschland) according to the manufacturer’s instruction. The RNA yield was assessed with a NanoDrop spectrophotometer, and 1 μg of total RNA was reverse transcribed using a Promega reverse transcription system (Promega, Madison, WI, USA) with random primers as recommended by the manufacturer. The resulting cDNA was used as the template for real-time amplification (LightCycler 2.0; Roche, Bâle, Suisse) using the specific primers (Table 2).

**TABLE 2:** Primers used for quantitative PCR using LightCycler

Primer	Sequence (5′–3′)	Reference
*gyrB* F	GGTGGCGACTTTGATCTAGC	Labandeira-Rey et al. (35)
*gyrB* R	TTATACAACGGTGGCTGTGC	Labandeira-Rey et al. (35)
*psmα1* F	TATCAAAAGCTTAATCGA	Li et al. (36)
*psmα1* R	CCCCTTCAAATAAGATGT	Li et al. (36)
*pmtB* F	CGTAGAGTCAAAGTGCCATATGGT	Joo et al. (30)
*pmtB* R	TGGGAATGATGATTGACTTAGAAGAA	Joo et al. (30)
*pmtR* F	GGACATGTTGCTCCTGGAGA	This study
*pmtR* R	GGTCCCTTTTCCTCTAATTGTTG	This study
RNAIII F	GGGATGGCTTAATAACTCATA	Labandeira-Rey et al. (35)
RNAIII R	GGAAGGAGTGATTTCAATGG	Labandeira-Rey et al. (35)

The relative amounts of toxin gene amplicons (*psmα*1, *hld* in RNAIII), for a part of Pmt, *pmtB*, for Pmt regulator, *pmtR*, were determined relative to an internal standard (*gyrB*) as described elsewhere (35). The expression levels of the investigated genes were therefore expressed as n-fold ratios compared to the standard in the presence of antibiotics relative to the same ratios in the absence of antibiotic (growth control). All quantitative PCR data were analyzed using the relative expression software tool REST 2009, version 2.0.13.

### Statistics.

Statistical analyses and graphs were performed using RStudio, version 0.99.893 (RStudio Team [2009–2016], integrated Development for RStudio, Inc, Boston, MA, USA). For each of the antibiotics conditions, the normality distribution of data (toxins release and mRNA fold change) was tested by shapiro test. If the data were normally distributed, conformity ttests were performed to compare the data under antibiotics conditions with the growth control (= 100% for toxins release, = 1 for mRNA fold change). For the antibiotics conditions without normally distributed data, conformity t tests were not performed and indicated as N.T. (not testable) in the figures. Comparison of the parameters between both clones were performed by the appropriate test according to normality of the data: nonparametric Wilcoxon test or parametric Welch test.
