# Supplementary material for: Sub-Inhibitory Concentrations of Oxacillin, but Not Clindamycin, Linezolid, or Tigecycline, Decrease Staphylococcal Phenol-Soluble Modulin Expression in Community-Acquired Methicillin-Resistant Staphylococcus aureus
Source: Microbiol Spectr. 2022 Jan 19;10(1):e00808-21. doi: 10.1128/spectrum.00808-21 (PMC8768629; doi:10.1128/spectrum.00808-21)
Supplement: SUPPLEMENTAL FILE 1 — Supplemental material. Download SPECTRUM00808-21_Supp_1_seq9.pdf, PDF file, 0.7 MB [file spectrum00808-21_supp_1_seq9.pdf]

Supplementary data

Figure S1: Effects of antibiotic sub-MIC on toxin release, A/ Hld, B/ PSMα1, at 6-h incubation for community-acquired methicillin-resistant *S. aureus* according to the clone (ST 80 European clone on the left, ST8 USA 300 on the right). The results for each antibiotic corresponding to different sub-MIC concentrations were pooled for the 2 strains belonging to the same genetic background. The results are expressed in percentage related to growth control without antibiotic (= 100%) represented by the dotted gray line. When possible, conformity t tests were performed to compare the expression with and without antibiotic. If not, N.D. is indicated. Comparison of HLD release between both clones for OXA performed using the Welch test. 1 point corresponds to 1 value (orange circle for LUG1799T; green triangle for ST20121288; blue square for SF8300; purple cross for HT20060752). CLI: clindamycin, LIN: linezolid, OXA: oxacillin, TIG: tigecycline, * $p < 0.05$, ** $p < 0.01$, *** $p < 0.001$. N.T.: not testable.

A

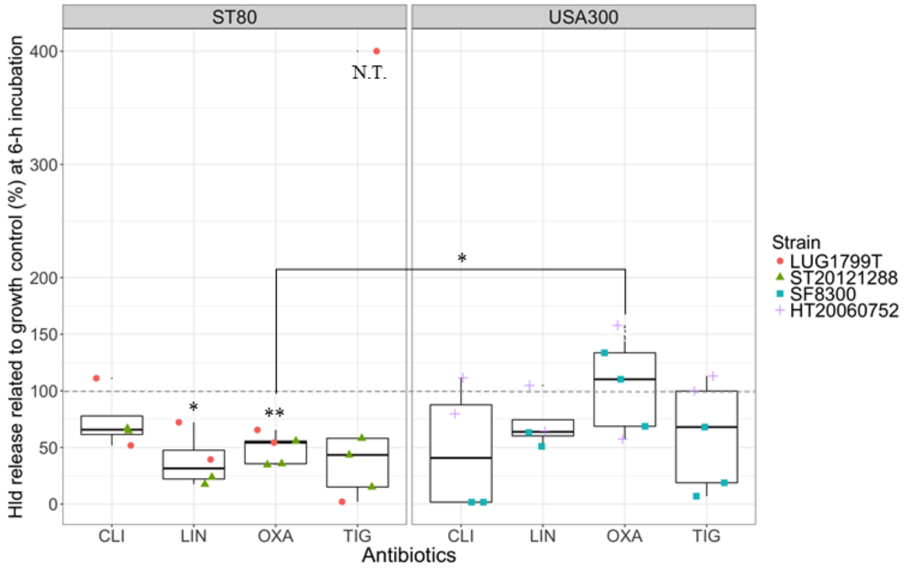

B

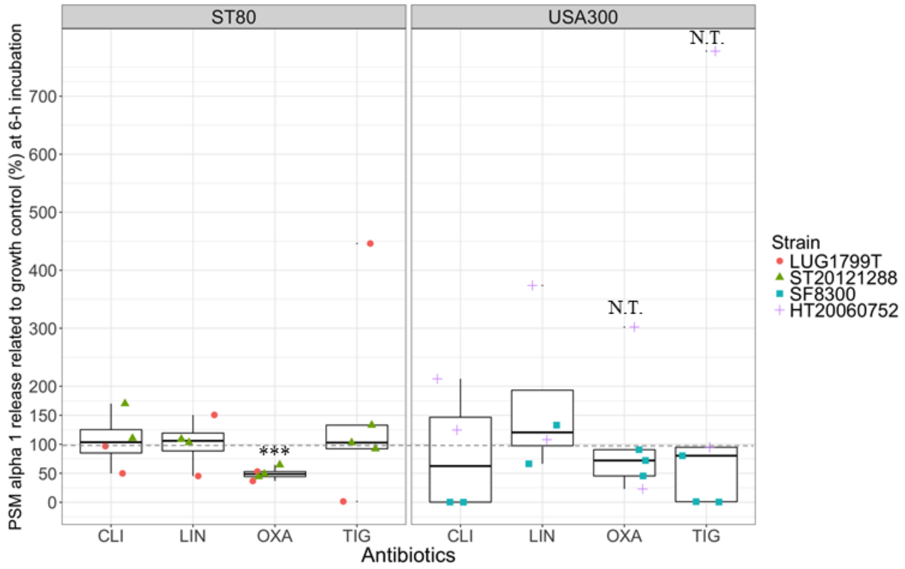

Figure S2: Effects of antibiotic sub-MIC on *psmα1* mRNA expression at 6-h incubation according to the clone (ST 80 European clone on the left, ST8 USA 300 in the right), pooling the different sub-MIC concentrations for one antibiotic. The results are expressed as n-fold differences in the psmα1/gyrb ratio in the presence of antibiotics relative to the control growth condition (i.e. without antibiotics, = 1) represented by the dotted gray line. When possible, conformity t tests were performed to compare the expression with and without antibiotics. If not, N.D. is indicated. 1 point corresponds to 1 value (orange circle for LUG1799T; green triangle for ST20121288; blue square for SF8300; purple cross for HT20060752). CLI: clindamycin, LIN: linezolid, OXA: oxacillin, TIG: tigecycline,* $p < 0.05$, N.T.: not testable.

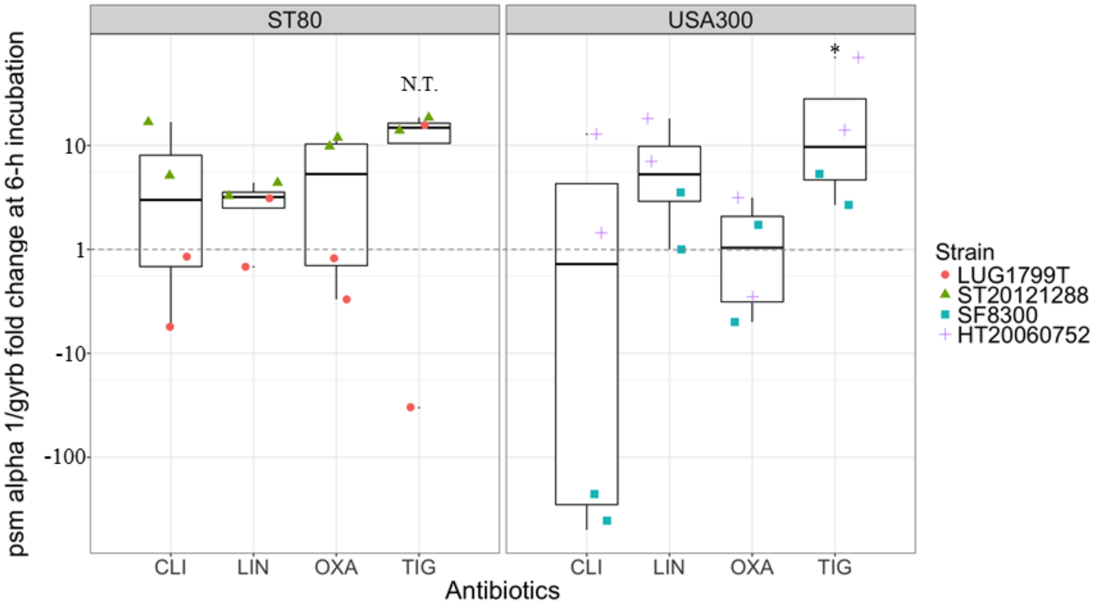

Figure S3: Growth curves for community-acquired methicillin-resistant *S. aureus*. Growth control: without antibiotics. A/ SF8300; B/ HT20060752; C/ LUG1799T; D/ ST20121288. CLI: clindamycin, LIN: linezolid, OXA: oxacillin, TIG: tigecycline, MIC = minimum inhibitory concentration.

A/

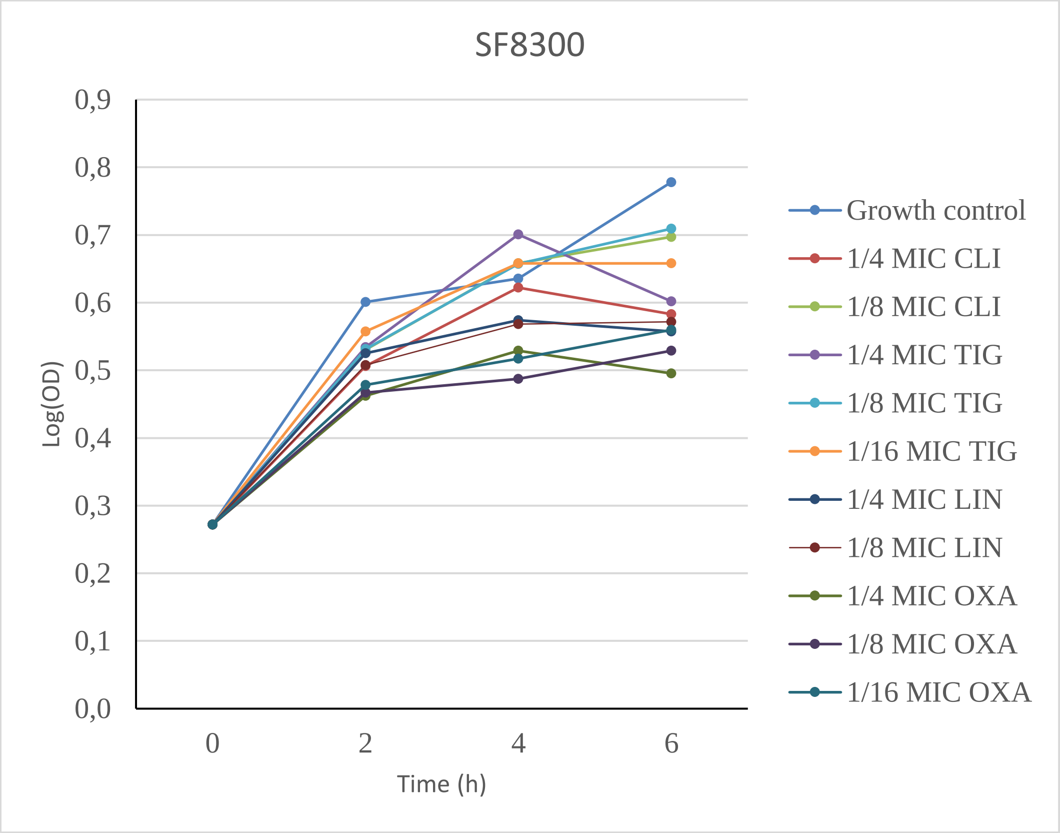

B/

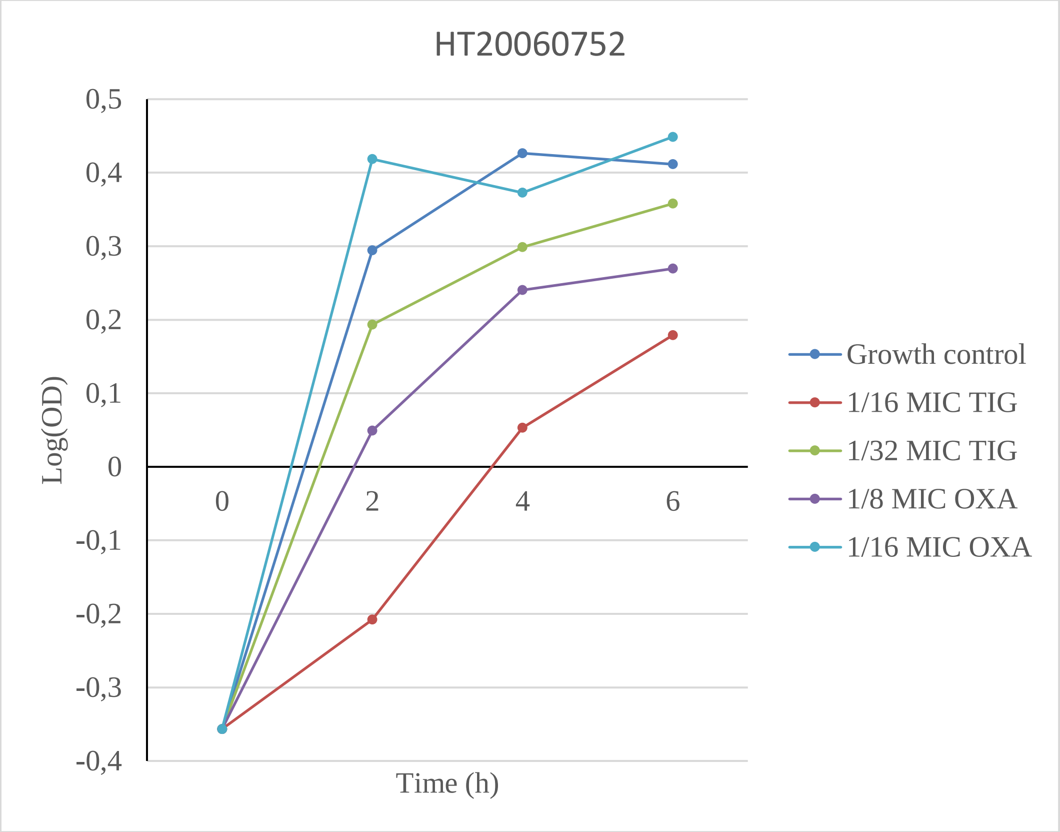

HT20060752
0,5
0,4
0,3
0,2
0,1
0
-0,1
-0,2
-0,3
-0,4
Log(OD)
0
2
4
6
Time (h)
Growth control
1/16 MIC TIG
1/32 MIC TIG
1/8 MIC OXA
1/16 MIC OXA

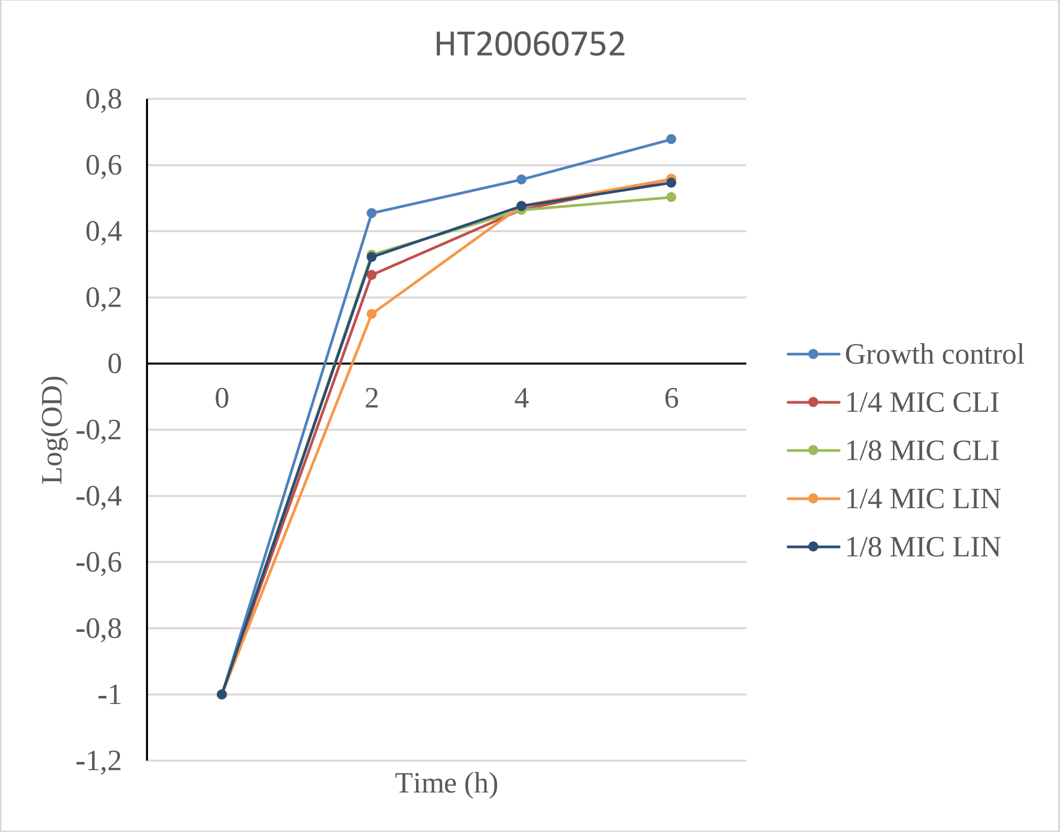

HT20060752
0,8
0,6
0,4
0,2
0
-0,2
-0,4
-0,6
-0,8
-1
-1,2
Log(OD)
0
2
4
6
Time (h)
Growth control
1/4 MIC CLI
1/8 MIC CLI
1/4 MIC LIN
1/8 MIC LIN

C/

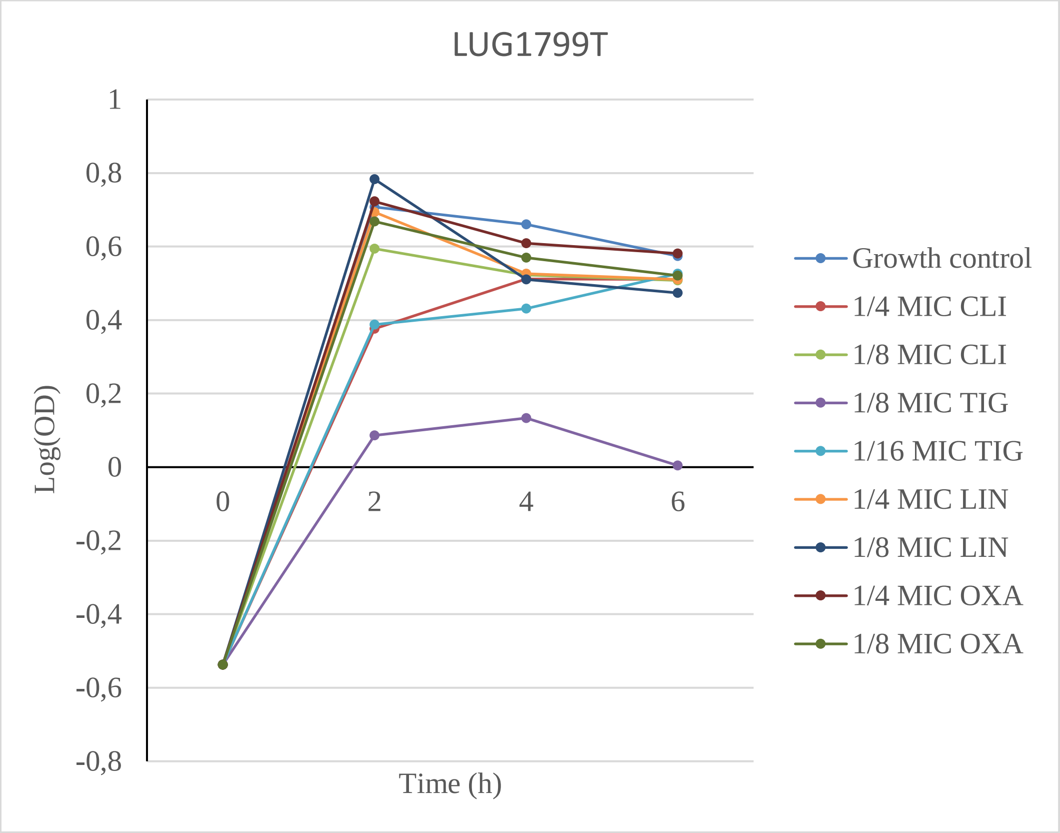
LUG1799T
1
0,8
0,6
0,4
0,2
0
-0,2
-0,4
-0,6
-0,8
Log(OD)
0
2
4
6
Time (h)
Growth control
1/4 MIC CLI
1/8 MIC CLI
1/8 MIC TIG
1/16 MIC TIG
1/4 MIC LIN
1/8 MIC LIN
1/4 MIC OXA
1/8 MIC OXA

D/

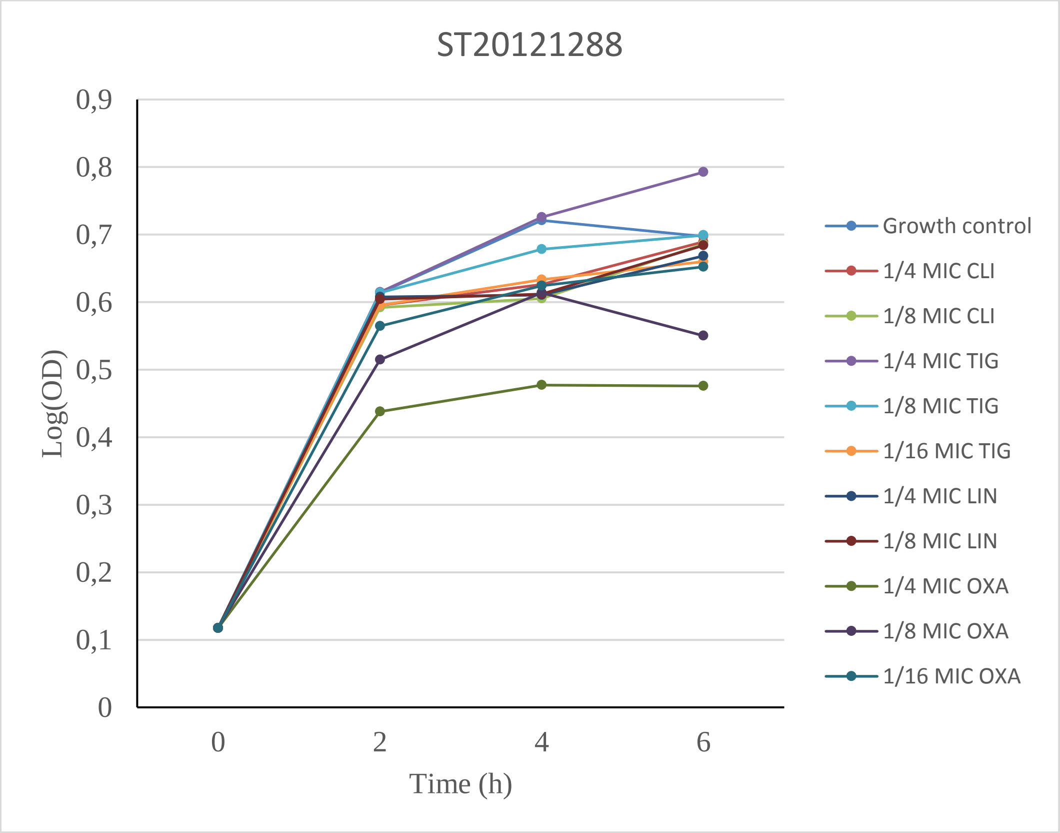
ST20121288
0,9
0,8
0,7
0,6
0,5
0,4
0,3
0,2
0,1
0
Log(OD)
0
2
4
6
Time (h)
Growth control
1/4 MIC CLI
1/8 MIC CLI
1/4 MIC TIG
1/8 MIC TIG
1/16 MIC TIG
1/4 MIC LIN
1/8 MIC LIN
1/4 MIC OXA
1/8 MIC OXA
1/16 MIC OXA
